# Cancer Pain Management in Resource-Limited Settings: A Practice Review

**DOI:** 10.1155/2011/393404

**Published:** 2011-12-11

**Authors:** Elizabeth Namukwaya, Mhoira Leng, Julia Downing, Elly Katabira

**Affiliations:** Department of Medicine, School of Medicine, Makerere University College of Health Sciences, P.O. BOX 7072, Kampala, Uganda

## Abstract

Pain in cancer is a common and burdensome symptom with different causes but in a significant number of cases it is undiagnosed and undertreated because of lack of skills for its assessment. Pain has significant negative impact on the patient and, therefore, it needs to be managed urgently and appropriately. In resource-limited settings, there are several barriers and challenges to pain management but even in these circumstances pain can be well managed with planned and innovative use of resources and if the World Health Organization public health system approach is used to ensure opioid availability.

## 1. Introduction

Pain in cancer patients is one of the most feared and burdensome symptoms [1] and is often undertreated [2]. In a recent systematic review, the prevalence of pain was found to be 64% in patients with advanced and metastatic disease, 59% in those on anticancer treatment, and 33% in those who had been cured of cancer [1]. In Africa, the prevalence of cancer pain ranges from 35.7% to 87.5% with the prevalence being more than 70% in most of the studies [3–9]. This high prevalence may be attributed to the limited treatment options for cancer, individuals presenting late, and comorbidity along with limited choice and availability of analgesics. In developed countries, evidence shows that 56–85% of cases of pain in cancer are tumor related, 5–62% of cases are related to cancer treatment, and 3–22% are unrelated to the cancer or its treatment [10, 11]. There is limited published data from resource-limited settings on the causes of pain in cancer patients; most data is limited to prevalence of pain in these patients and in most cases it is assumed that pain is due to the tumor [3–9, 12, 13]. This is probably because in resource-limited countries there are limited treatment options for most cancers, and patients present late with advanced malignancy, and, therefore, it is likely that more of the pain will be due to the tumor and less due to treatment as compared to developed countries. Pain unrelated to the cancer or its treatment is of significant importance in resource-limited settings, where the burden of HIV/AIDS is high and, therefore, patients might have HIV-associated malignancies and their pain may be due to the HIV or associated opportunistic infections and/or antiretroviral treatment.

 Therefore, a good pain assessment is important to identify the cause and be able to manage it effectively. 

## 2. The Impact of Cancer Pain on the Patient

Chronic cancer pain has a significant impact on the patients and their family. Thirty-six percent of patients on anticancer treatment have reported their pain to be of moderate-to-severe intensity and 45% of patients with advanced, terminal, and metastatic disease reported pain of moderate-to-severe intensity in a systematic review done on studies carried out mainly in developed countries [1]. In resource-limited settings, pain has been found to be of moderate-to-severe intensity in 30% to 70% of cases. In the majority of the studies, moderate-to-severe pain was registered in more than 50% of the patients [3, 13–16].

An individual's perception of pain may be influenced by many issues including psychological, social, cultural, and spiritual factors. Koffman et al. explored the cultural meanings of pain in White British and Black Caribbean. They found that the Black Caribbean had a higher prevalence of pain and reported more refractory pain than the White British. Specific to the Black Caribbean population in this study was the belief that pain was a test of faith and a punishment. Therefore, patients may be able to accommodate distress depending on what meaning is held about the pain [17]. In some cultures in Africa, it has been observed that children and adults will not complain of pain or express it because it is a cultural norm not to appear weak and to show consideration for family members and health professionals [18, 19]. In the authors' experience, most adults in Africa will deny or underreport their pain so that they do not discourage their health care and nonhealth care carers. Also they will underreport pain because it is expected of them from the society to bear some pain. This may be one of the factors contributing to the lower dosages of opioids used as observed in Uganda.

Unrelieved pain is associated with unnecessary suffering, functional impairment, affects sleep and appetite, leads to psychological and spiritual distress, and may lead to rejection of a potentially beneficial treatment modality if it leads to pain which may impact one's survival [20, 21]. Severe pain that affects one's performance of activities of daily living and enjoyment of life, has been found to be a predictor of anxiety and depression [22].

## 3. Pain Assessment and Management

Pain assessment is a prerequisite for the diagnosis and appropriate management of pain. However, deficiencies in pain assessment and management have been found in several settings where cancer patients are seen [23]. In many resource-limited settings, there are few oncologists and palliative care specialists and many cancer patients may be treated by Health Professionals without the skills of pain assessment because there was no provision for palliative care in their training. Some health professionals pay more attention to the disease thinking that disease control alone will lead to pain relief [12]. Assessment of pain is an ongoing process, which starts with a comprehensive evaluation of the patient's pain, symptoms, functional status, and clinical history, dependent upon the patient's presenting needs. When assessing an individual's pain, it is important to not manage the pain in isolation and to treat the underlying cause where possible [24].

## 4. Management of Cancer Pain

The control of pain and suffering is central to health, and the right to health is stipulated in several International declarations [25]. The Korean declaration states that ^“^ *Every individual has the right to pain relief” *[26]. The Cape Town Declaration in 2002 stated that the control of pain and symptoms is a human right, and, therefore, appropriate drugs should be available in every country in sub-Saharan Africa as part of the essential drug list, including opioids such as morphine. These drugs should be available and accessible at all levels including the community [27]; however, this is often not the case [28]. The human rights initiative advocates for pain relief as a human right and the denial of pain relief or the nonaccessibility of palliative care as an abuse of this right [29]. In 70–90% of people with cancer pain, the World Health Organization (WHO) approach to pain management which emphasizes the principles of “by the clock, by the mouth, by the ladder, and by the individual” is sufficient for pain control [30].

**Figure 1:**
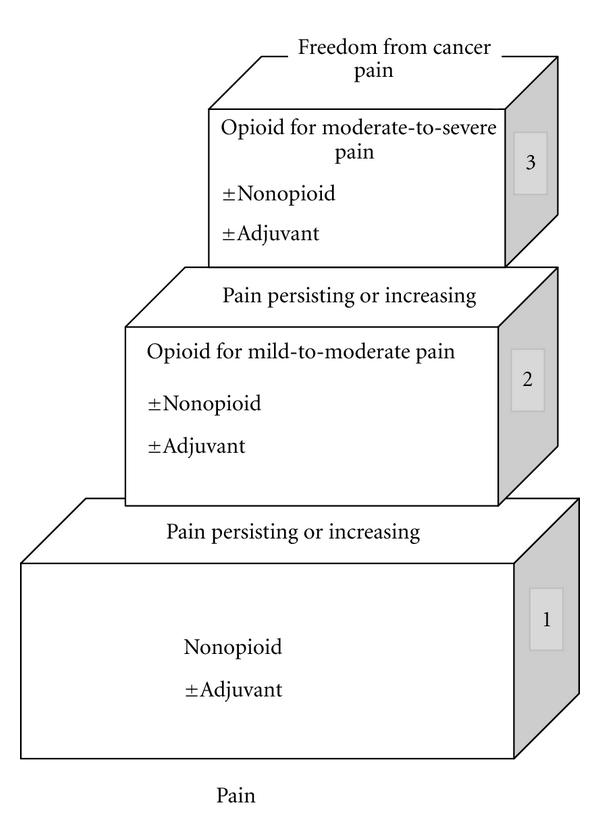
WHO analgesic ladder.

The analgesic ladder (Figure 1) is a stepwise approach to the management of pain based on the severity and the type of pain [30].

Mild pain is treated using nonopioids such as paracetamol and nonsteroidal anti-inflammatory drugs (NSAIDS-Step-1); if mild pain is not controlled or if the pain is more severe, opioids for mild-to-moderate pain should be used in addition to the NSAIDs (Step 2). Severe or moderate pain, or pain not controlled by Step 2 analgesics, is treated with strong opioids (Step 3), see Figure 1. Adjuvant analgesics should also be used at any of the steps if appropriate, for example, for bone pain, nerve pain, or pain not fully relieved by opioids, and laxatives should be given for patients on opioids to prevent constipation and other side effects should be managed. This 3-step ladder is currently being challenged as in some parts of the world, for example, Africa, step 2 medications are expensive and practice has developed that uses low-dose oral morphine (Step 3) for moderate pain. This may prove to be an effective and efficient model and is an ongoing subject for research. The 2-step ladder is being recommended in the new guidelines for the management of persistent pain in children, along with a call for research in this area [31].

Despite the success of the WHO analgesic ladder, much cancer pain remains uncontrolled. The prevalence of undertreatment of pain has been found to be as much as 43% [32]. There are many barriers to effective pain control, and these include a lack of national policies and low priority assigned to pain relief; a lack of national standards and guidelines for providing pain control medications; legal restrictions on the importation and medical use of opioids; the greatly exaggerated concern that the use of opioids will lead to drug abuse and addiction; lack of awareness by health professionals and the public including families who care for the dying at home that cancer pain can be relieved; social stigma surrounding cancer which delays or prevents adequate treatment of the disease and pain [33].

The low priority assigned to pain relief is reflected in the few palliative care services that are available in many resource-limited countries. For example, India with a population of 1.2 billion, has just a few localized services with the exception of the state of Kerala[34].

Areas of longstanding conflict, political instability, or natural disaster face the biggest challenges for health care in general, and palliative care in particular. Drug availability is also a major challenge for pain control in resource-limited settings. Opioids are key in the management of severe pain, and morphine consumption is a proxy measure of the degree of pain control. Morphine and codeine are both considered to be essential drugs and appear on the WHO essential drugs list. Essential drugs are defined as those that satisfy the health care needs of the majority of the population, that should be available all the time in adequate amounts and in appropriate dosages [35]. However, despite this, opioid availability is very limited in many resource-limited countries. In 2007, seven developed countries accounted for more than 80% of global morphine consumption, while developing countries which represent more than 80% of the world's population accounted for less than 10% [36]. In India, it is estimated that less than 0.4% of the 1.2 billion population has access to oral morphine [34].

In a survey done in 12 African countries looking at the availability of pain-relieving medications, factors that affected opioid availability included (Table 1) issues related to policy, clinical issues, and resource issues [35].

 Thus the provision of pain control for people with cancer in low resource settings poses a challenge for all involved.

## 5. Strategies That Have Been Used in Resource-Limited Settings to Improve Pain Control

**Figure 2:**
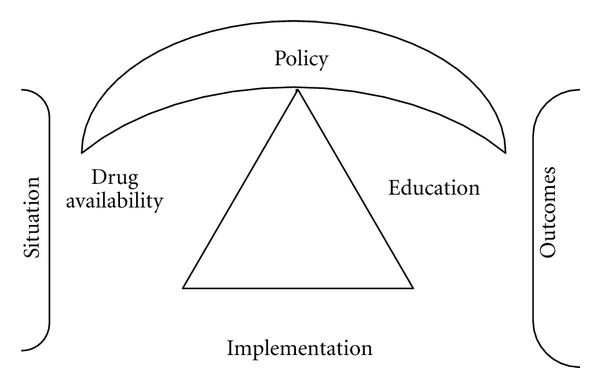


A public health systems approach as recommended by the WHO is essential in addressing issues of availability and access to opioids and ultimately pain control. In this approach, there are four key foundation measures in ensuring sustainable palliative care services. These include a government policy, drug availability, education, and implementation (Figure 2) [37].

### 5.1. Policy

The WHO recommends that all governments evaluate their policies and procedures to ensure that while opioids are controlled to prevent drug abuse and drug trafficking they are received by patients requiring analgesia [38]. Established in 1996, the Pain and Policy Studies Group (PPSG) has played an important role in evaluating national reports and policies and has worked in collaboration with palliative care professionals and governments in many parts of the developing world. It examines trends, identifies barriers, and seeks to develop plans to improve the availability of opioids [39].

In some resource-limited countries, such as Uganda, addressing the foundation measures has been crucial in developing sustainable palliative care services and has enhanced pain control. In Uganda, there was extensive advocacy to health officials, health planners, health care professionals, pharmacists, political leaders, and the community for several years. Following this a stakeholder's workshop was convened in 1998 and a task force established to draft a national policy for palliative care. The Ministry of Health at the same time was discussing the first Health Service Strategic Plan (HSSP) for the years 2000–2005. Through further advocacy and WHO support, the Palliative Care Policy was incorporated into the HSSP, and palliative care was put in the package of essential clinical services to be delivered in government health institutions, for the first time [40]. The policy provides for the provision of palliative care drugs including pain medication, capacity building for palliative care, strengthening referral systems for palliative between health services and home care, and community-based rehabilitation of the terminally ill [40].

In India, PPSG worked in partnership with the Indian palliative care workers and the central and state governments to bring about reforms in the policies including exempting all palliative care centres from the need for a drug licence, which freed palliative care centres from the need to have a pharmacist. The government of India in 1998 instructed all state governments to amend and simplify their narcotics regulations [39]. The Government of India has also included palliative care in the National Cancer Control Programme 5-year plan which began in April 2007. However, changes in policy alone have not been sufficient to improve opioid availability in this country. The experience in India, therefore, stresses the need for a comprehensive public health approach for palliative care [34].

### 5.2. Drug Availability

To ensure drug availability, it is not enough just to address policy barriers, there should also be an effective distribution network and there is a need to remove other barriers such as a poor distribution system and lack of trained personnel which leads to lack of accessibility. Poorly developed health systems are, therefore, a significant limiting factor with the growth of palliative care linked to indices of human development and economic strength [41].

Lack of health care workers able to dispense and prescribe is a major issue in Africa as prescribing is limited to those with special licenses or training (commonly doctors), and yet there are very high patient-to-doctor ratios [41]. An innovative approach was taken to this obstacle in Uganda by an amendment to the existing narcotics legislation to allow specialist palliative care nurses and clinical officers to prescribe morphine [42]. Uganda was the first country to make this important step. Currently, these nurses and clinical officers complete a nine-month, full-time, Clinical Palliative Care Course (CPPC) or a bachelor's degree in Palliative Care, and both of these programs include theoretical learning, practical skills training in a number of clinical settings, and exposure to a public health planning approach. These graduates then are able to prescribe morphine in the different districts in Uganda. This highly successful model is being closely watched by several other countries, and an evaluation of the programme is planned; however, it may offer a major advance in morphine accessibility [41].

### 5.3. Education

Education in palliative care is paramount in ensuring a change in culture, especially to address the barrier of health professionals' lack of knowledge and skills for pain management and to enhance the right attitudes to palliative care practices. Although regulatory barriers and availability issues may be addressed, there may be little use of opioids because of health professionals' associated barriers [34, 43].

 One of the strategies used in Uganda to ensure education in palliative care was to introduce it in the medical students and nurses curriculum in 1993 and recently in the curriculum of some of the postgraduate courses. In addition to the CPCC course that allows nurses to prescribe morphine, there is a diploma and degree course in palliative care offered in Uganda for all Africa. There are also short courses for health professionals, allied health professionals, community volunteers, and traditional healers.

### 5.4. Implementation

In implementation, it is important to identify opinion leaders in the community and in health care organizations that have potential of becoming centers of excellence in palliative care based on their leadership potential then develop a plan to support the evolving program with the goal of providing care for all [37]. In Uganda, involving the Deans of medical schools, tutors in nursing schools, and hospital administrators has been key in ensuring that palliative care was introduced in the doctors and nurses' curricula and in the cases of those who have already qualified and are working, to ensure training in a health professionals' course in palliative care. After their training, all the different trained health professionals go to different parts of the country and with these skills are able to manage pain with a wide coverage [40]. The neighborhood Network in Palliative care Initiative in Kerala India demonstrates successful community involvement in palliative care [44].

In resource-limited settings, it is not always possible to use the WHO 3-step ladder approach to pain control because of the limited availability of opioids for mild-to-moderate pain (Step 2). These are not usually available in the government institutions and are expensive on the open market. Morphine powder reconstituted into morphine solution has been found to be cheaper than step 2 opioids and, therefore, in Uganda, for example, step 2 analgesia is provided by giving lower doses of morphine which are equivalent to step 2 analgesia. There is also a challenge in giving analgesia on the clock when some of the population in the rural areas have no clocks. Some palliative care services in Uganda have designed a treatment card guiding patients on when to take their drugs using the sun rising and setting.

Getting the appropriate dosage for those who live very far from the health care services and will not be reviewed in a short time is also another challenge. In India, research has shown that the daily oral morphine requirement can be determined by giving small boluses of 1.5 mg of intravenous morphine at short intervals of about every 10 minutes until pain is controlled or until the patient becomes drowsy and the oral four-hour dose needed was found to be equal to the total initial intravenous requirement [45].

Another challenge is the patient who is dying or is unconscious and is in pain, given the limited facilities or lack of, thereof, subcutaneous use of opioids. In Uganda, higher concentrations of morphine in small amounts of liquid are given to be absorbed buccally, and in India, families have been taught to use the subcutaneous morphine at home.

Pain unresponsive to the analgesic ladder is a challenge in resource-limited settings where anesthetic techniques may be out of reach because of the limited number of anesthetists and because they are expensive. The use of the intravenous formulation of ketamine orally for control of pain unresponsive to the WHO analgesic ladder has been studied in small studies in several settings [46]. Ketamine is cheap and readily available and is worth considering for resource-limited countries.

## 6. Conclusion

Cancer pain of moderate-to-severe intensity is common in resource-limited settings and impacts the sufferers' quality of life. Pain relief is a human right which should be observed for everyone. There are several challenges in pain management in resource poor settings but these can be overcome through the adoption of the WHO public health systems approach to integrating palliative care services. This involves having a government policy on pain management and ensuring opioid availability and accessibility. It also means educating health care professionals on pain assessment and management as well as mobilizing and empowering the public on the need to control pain. Given the limited resources and overwhelming need, local champions have a crucial role in developing and implementing innovative solutions underpinned by a rich evidence base. Quality of care needs to be considered alongside increasing coverage. Crucial to this approach will be integration to strengthen health systems and ensuring pain control becomes embedded in models of delivery of health care. There is a need for being innovative and maximizing use of available use of resources in pain management in these settings.

## Figures and Tables

**Table 1:** Factors affecting opioid availability in 12 African countries.

Policy issues	Clinical issues	Resources
-Tight regulations on opioids -No national policy -Bureaucratic process for legislation -Punitive regulations -Inadequate ability to report consumption to the narcotics board -Lack of political will	-Limited time for prescription -Lack of knowledge and skills by health professionals to assess and manage pain -Fear of addiction -Lack of enough prescribers	-Unreliable stocks -Few dispensers -Lack of storage for opioids
